# Comparison of Two Pulsed Field Ablation Systems for Atrial Fibrillation: One‐Year Outcomes From a Multicenter Registry

**DOI:** 10.1111/jce.70375

**Published:** 2026-05-26

**Authors:** Marisa van der Graaf, Bob G. S. Abeln, Jippe C. Balt, Suneet Mittal, Bradley P. Knight, Melanie A. Gunawardene, Bradley Wilsmore, Amardeep Amardeep, Tim Harloff, Max Liebregts, Michael M. Malaty, Dan L. Musat, Graham Peigh, Supreet Singh, Maurits C. E. F. Wijffels, Stephan Willems, Nishant Verma, Vincent F. van Dijk, Lucas V. A. Boersma

**Affiliations:** ^1^ Department of Cardiology St. Antonius Hospital Nieuwegein the Netherlands; ^2^ Department of Cardiology Amsterdam UMC Amsterdam the Netherlands; ^3^ Snyder Center for Comprehensive Atrial Fibrillation Valley Health System Paramus New Jersey USA; ^4^ Division of Cardiology, Feinberg School of Medicine Northwestern University Chicago Illinois USA; ^5^ Department of Cardiology and Intensive Care Medicine Asklepios St. Georg Clinic Hamburg Germany; ^6^ Department of Cardiology John Hunter Hospital New Lambton New South Wales Australia

**Keywords:** atrial fibrillation, long‐term efficacy, pulmonary vein isolation, pulsed field ablation

## Abstract

**Background:**

While various pulsed field ablation (PFA) systems for atrial fibrillation (AF) ablation have become available in recent years, data comparing long‐term efficacy outcomes remain limited.

**Purpose:**

To compare long‐term efficacy outcomes of two commercially available PFA systems to perform pulmonary vein isolation (PVI) for AF in a multicenter clinical setting.

**Methods:**

We conducted an international, multicenter, registry study of patients with AF undergoing a first ablation between January 29, 2024, and September 1, 2024. Patients were treated with either the pentaspline catheter or the circular over‐the‐wire catheter in all centers. Endpoints included time to recurrence of atrial arrhythmias following a 2‐month blanking period and repeat ablation outcomes. Factors associated with recurrence were assessed.

**Results:**

A total of 428 patients were included, of whom 231 (54.0%) were treated with the pentaspline catheter. Most patients (84.6%) underwent a PVI‐only procedure, with significantly longer procedure and LA dwell times observed in the circular catheter group (*p* < 0.001). Acute procedural success was achieved in all patients. At 12 months, AF‐recurrence was observed in 33.5% of patients overall, with similar rates between groups (pentaspline catheter 34.1% vs. circular catheter 32.8% [log‐rank *p* = 0.93]). A longer time since AF diagnosis and persistent AF were associated with recurrence of AF. Repeat ablation was performed in 14.9% of patients.

**Conclusion:**

In this study, we compared two commercially available PFA systems for primary PVI in AF patients. Both achieved high acute success and similar 12‐month AF recurrence rates, indicating comparable efficacy.

## Graphical abstract

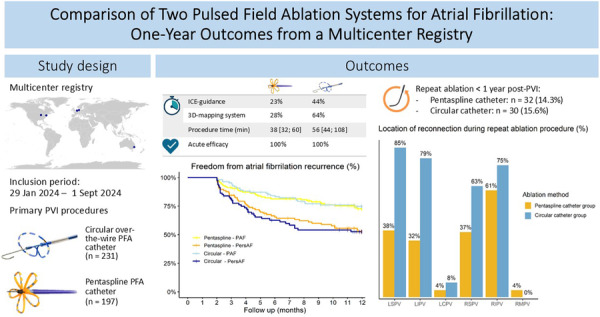

**Abbreviations**
AF: atrial fibrillation
CIED: cardiac implantable electronic device
ICE: intracardiac echocardiography
LA: left atrium
LAVI: left atrial volume index
LVEF: left ventricular ejection fraction
PFA: pulsed field ablation
PV: pulmonary vein
PVI: pulmonary vein isolation
TEE: transesophageal echocardiography

## 1 Introduction

Catheter‐based pulmonary vein isolation (PVI) ablation procedures have become the cornerstone for treatment in patients with symptomatic atrial fibrillation (AF) [1]. As the prevalence of AF continues to rise, the need for safe, fast, and efficient catheter ablation techniques has become more pressing. Pulsed field ablation (PFA) is taking the ablation market by storm as it fulfills this unmet need [2]. Due to its relative tissue selectivity, PFA further reduces the risk of complications associated with thermal ablation techniques, including permanent phrenic nerve injury, pulmonary vein stenosis, and atrio‐esophageal fistula [3, 4, 5, 6]. Furthermore, short‐term efficiency outcomes seem to be comparable to thermal ablation outcomes, and PVI using PFA is characterized by short, efficient procedures and a steep learning curve [7, 8]. Various PFA systems have become commercialized, that differ in aspects such as catheter design and pulse profile, which can influence both efficacy and safety outcomes [9]. Although short‐term efficacy and safety outcomes have been published, data on long‐term efficacy outcomes remain limited. Additionally, studies comparing long‐term efficacy outcomes between different PFA systems are currently lacking. This is, however, important information for identifying the safest and most effective PFA platform. In this study, we evaluated and compared the long‐term efficacy outcomes of two commercially available PFA systems in performing PVI for AF in a real‐world, multicenter clinical setting, in centers where both the Pentaspline and Circular array PFA systems are commonly used.

## 2 Methods

### 2.1 Trial Design

We performed an international, multicenter, observational registry study that evaluated the safety and efficacy of the FARAPULS, pentaspline catheter PFA system (Boston Scientific Inc., MA, USA) and PulseSelect, circular over‐the‐wire catheter PFA system (Medtronic, MN, USA). A total of five centers participated in this study: Asklepios St. Georg Clinic (Germany), John Hunter Hospital (Australia), Northwestern Memorial Hospital (USA), St. Antonius Hospital (The Netherlands), and Valley Health System (USA). At each center, the local ethics committee approved the collection of the required data. The study was performed in accordance with the Declaration of Helsinki.

### 2.2 Study Participants

Data from consecutive patients undergoing a first PVI using PFA for paroxysmal or persistent AF between January 29 and September 1, 2024, were collected. In each center, both PFA systems were used side‐by‐side during the inclusion period. Patients were not selected for a specific PFA system, and assignment was independent of patient characteristics and operator preference. Patient data were prospectively entered into each center's local ablation database and then merged for retrospective analysis. Patients with a prior left atrial ablation procedure were excluded from the study. Data collection included registration of baseline and procedural characteristics, periprocedural safety outcomes, the occurrence of adverse events, information regarding the use of anti‐arrhythmic drugs during a 12‐month follow‐up period, atrial arrhythmia recurrence up to 12 months post‐procedure following a 2‐month blanking period, and repeat ablation outcomes.

### 2.3 Ablation Procedure and Follow‐Up

The ablation procedures were performed per local institutional standards and in accordance with the instructions for use of the ablation systems. Detailed information regarding differences in institutional standards and procedural characteristics were published before [8]. In short, procedures were performed with either fluoroscopy guidance, intracardiac echocardiography, and/or a 3D mapping system as per hospital standard. All pulmonary veins were targeted with the PFA system, while additional structures could be targeted with the PFA system according to operators' discretion.

Based on patient's symptoms and the discretion of the treating physician, anti‐arrhythmic drugs and anticoagulation therapy could be discontinued or changed during follow‐up. Rhythm monitoring during outpatient clinic visits could be performed with the use of either 12‐lead ECG‐ and Holter‐recordings or interrogation of cardiac implantable electronic devices (CIED). In addition to standardized follow‐up visits, the St. Antonius hospital offers participation in a remote monitoring program with the use of photoplethysmography (PPG)‐based recording systems. All recorded atrial arrhythmic events of at least 30 s, after a 2‐month blanking period, were considered recurrence of arrhythmia. In the case of symptomatic arrhythmia events after the blanking period, patients were eligible for a redo‐ablation procedure at the operator's discretion.

### 2.4 Endpoints

The primary efficacy endpoint was defined as the recurrence of AF at 12 months follow‐up, after a 2‐month blanking period. Secondary endpoints included procedural characteristics, the recurrence of atrial arrhythmias (AF, atrial flutter [AFL], or atrial tachycardia) during 12 months of follow‐up, major adverse events, the incidence of repeat ablation procedures, and the number of PVs with reconnection during repeat ablation procedures. Subanalyses were performed to assess the relationship between baseline and procedural characteristics and the primary and secondary endpoints.

### 2.5 Data Analysis and Statistics

Since this was an observational registry and given the limited availability of outcome data at the start of the commercial use of the circular array, no formal hypothesis was tested, and no power calculation was made for the study. Safety outcomes were analyzed in all patients who underwent the ablation procedure, while analyses for arrhythmia outcomes were only performed in patients with at least 2 months of follow‐up. Data analysis was conducted at the St. Antonius Hospital using R, version 4.4.0 (R Foundation, Vienna, Austria). Variables were presented as mean ± SD or median [IQR] for continuous variables, and frequencies with percentages for categorical variables. Continuous variables were compared using the Student's *t*‐test or Mann−Whitney *U* test; categorical variables were compared using the chi‐square test or Fisher's exact test. Kaplan−Meier curves and Log‐rank tests were used to determine and compare the incidence and time‐to‐event of efficacy outcomes. In case patients were lost to follow‐up, data were censored on the date the last rhythm monitoring was performed. Patients with complete 1‐year follow‐up data were censored at 365 days post‐procedure. Univariate and multivariable Cox‐regression analyses were conducted to compare the performance of the different PFA‐systems, adjusting for known predictors of arrhythmia recurrences, including, amongst others: age, female sex, type of AF, the use of mapping systems during procedure, and left atrial size. Given the study design, it could not be assumed that missing data were “missing completely at random”; therefore, no imputation was performed [10]. Multivariable Cox regression analysis included all variables with a *p* ≤ 0.10 in the univariate analyses, along with the ablation system. For variables with missing data in ≥ 25% of patients, with significant association with the primary endpoint in univariate analyses, two approaches were applied: (1) a complete‐case multivariable analysis including the variable, and (2) an alternative multivariable analysis excluding the variable. Procedure duration and other continuous procedure‐related outcomes were assessed when applicable using multivariable linear regression. All reported *p* values are two‐sided, and a significance level *α* of 0.05 was used.

## 3 Results

A total of 428 consecutive patients undergoing a primary PFA PVI between January 29, 2024, and September 1, 2024, were included in this analysis. The pentaspline catheter was used in a total of 231 (54.0%) patients, while the remainder (*n* = 197, 46.0%) were treated with the circular catheter.

### 3.1 Baseline

Baseline characteristics for the overall population and stratified per ablation system are presented in Table 1. In brief, the majority of patients were male (*n* = 285, 66.6%) with a mean age of 63.0 ± 10.4 years. Most patients had paroxysmal AF (*n* = 242, 56.5%) with an EHRA‐score of 2 (85.0%) at the time of the ablation and a mean CHA_2_DS_2_‐VA‐score of 1.5 ± 1.5. Echocardiography outcomes within 1 year before procedure (*n* = 309) showed a mean LAVI of 33.2 ± 10.4 mL/m and a reduced LVEF ( < 40%) in 13 patients (4.1%). The use of class I or III anti‐arrhythmic drugs was higher in the pentaspline catheter group (*n* = 143 [61.9%] vs. *n* = 83 [42.1%], *p* < 0.001), while β‐blockers were used less often in the pentaspline catheter group: 96 patients (41.6%) versus 106 patients (53.8%), *p* = 0.015.

**Table 1:** Baseline characteristics.

	Pentaspline catheter group (*n* = 231)	Circular catheter group (*n* = 197)	*p* value
Female sex	77 (33.4)	66 (33.5)	1.000
Age, years	61.6 ± 10.5	64.7 ± 10.0	0.002
BMI, kg/m^2^	28.0 ± 5.4	28.1 ± 4.6	0.855
Hypertension	94 (40.7)	78 (39.6)	0.895
Diabetes mellitus	22 (9.5)	25 (12.7)	0.374
Heart failure	27(11.7)	26 (13.2)	0.745
OSA	36 (15.6)	29 (14.7)	0.910
CHA₂DS₂‐VA‐score	1.3 ± 1.3	1.7 ± 1.6	0.003
CHA₂DS₂‐VA‐score categories			0.071
0	80 (34.6)	50 (25.4)	
1	64 (27.7)	54 (27.4)	
2+	87 (37.7)	93 (47.2)	
LAVI, mL/m^2^ (*n* = 225)	32.9 ± 10.7	33.6 ± 10.1	0.592
LVEF < 40% (*n* = 317)	10 (6.0)	3 (2.0)	0.133
Type of AF			0.687
Paroxysmal AF	128 (55.4)	114 (57.9)	
Persistent AF	101 (43.7)	80 (40.6)	
Longstanding persistent	2 (0.9)	3 (1.5)	
Time since AF diagnosis, years	1.6 [0.6, 4.9]	1.6 [0.5, 4.5]	0.289
EHRA symptom classification			0.237
Class 1 (No)	7 (3.3)	3 (1.7)	
Class 2 (Mild)	175 (82.5)	154 (88.0)	
Class 3 (Severe)	27 (12.7)	18 (10.3)	
Class 4 (Disabling)	3 (1.4)	0 (0.0)	
Preprocedural medication			
Beta‐blocker	96 (41.6)	106 (53.8)	0.015
Class I or III antiarrhythmic	143 (61.9)	83 (42.1)	< 0.001
Anticoagulant therapy, DOAC	225 (97.4)	186 (94.4)	0.184
Anticoagulant therapy, VKA	3 (1.3)	2 (1.0)	1.000

*Note:* Data is presented as counts (percentages) for categorical data, means ± standard deviations for normally distributed continuous data, and median [interquartile range] non‐normally distributed continuous data.

Abbreviations: BMI, body mass index; DOAC, direct oral anticoagulant; LAVI, left atrial volume index; LVEF, left ventricular ejection fraction; OSA, obstructive sleep apnea; VKA, vitamin K antagonist.

### 3.2 Procedure

The majority of patients (*n* = 362, 84.6%) underwent a PVI‐only procedure in both treatment groups, with additional ablation mostly focused on posterior wall isolation in both treatment groups (pentaspline catheter group: *n* = 35 [15.2%] vs. circular catheter group: *n* = 30 [15.7%], *p* = 0.974). Additional imaging modalities were used significantly less often during procedures with the pentaspline catheter compared to the circular catheter (see Table 2). The total number of applications did not differ between groups; however, patients in the pentaspline catheter group more frequently received > 8 applications in the LSPV compared with the circular catheter group (44.9% vs. 26.0%, *p* < 0.001). The total procedure time was shorter in the pentaspline catheter group compared to the circular catheter group: 38.0 [32.0; 60.2] versus 55.5 [44.0; 107.8] min, respectively (*p *< 0.001). Multivariable linear regression showed that use of the circular catheter (+18%), general anesthesia (+25%), the use of a 3D‐mapping system (+58%), and ICE‐guidance during procedures (+76%) were all independently associated with longer total procedure time. On the other hand, PVI‐only procedures were associated with shorter procedure times (12%). In addition, the LA‐dwell time was shorter in the pentaspline catheter group: 27.0 [23.0; 43.5] versus 45.5 [34.0; 88.2] min, *p* < 0.001. The multivariable analysis of LA‐dwell time demonstrated generally similar results to the total procedure time analysis. Results from both multivariable linear regression analyses are shown in Supporting Information S1: Table 1. Vascular closure was performed with the use of a figure of eight suture in the majority of patients in both groups (pentaspline catheter: *n* = 182 [78.8%] vs. circular catheter: *n* = 154 [78.2%], *p* = 0.971), while a closure device was used in the remainder of patients. Protamine was used in 79 (34.2%) versus 45 (22.8%) patients in the pentaspline catheter group and circular catheter group, respectively (*p *= 0.013) (Table 2). Acute procedural success, defined as the successful isolation of all ablation targets, was achieved in all patients.

**Table 2:** Procedure characteristics.

	Pentaspline catheter group (*n* = 231)	Circular catheter group (*n* = 197)	*p* value
General anesthesia (instead of conscious sedation)	207 (89.6)	126 (64.0)	< 0.001
Ultrasound‐guided vascular access	210 (90.9)	164 (83.2)	0.026
Use of echocardiography—TEE	4 (1.7)	23 (11.7)	< 0.001
Use of echocardiography—ICE	54 (23.4)	86 (43.7)	< 0.001
Electroanatomic mapping system	64 (27.7)	126 (64.0)	< 0.001
PVI‐only procedure	196 (84.8)	166 (84.3)	0.974
Number of applications			
LSPV	8.0 [8.0, 10.0]	8.0 [8.0, 10.0]	0.020
LIPV	8.0 [8.0, 8.0]	8.0 [8.0, 8.0]	0.965
LCPV	16.0 [16.0, 17.0]	16.0 [15.0, 16.5]	0.679
RSPV	8.0 [8.0, 10.0]	8.0 [8.0, 9.0]	0.007
RIPV	8.0 [8.0, 10.0]	8.0 [8.0, 9.0]	0.185
RMPV	8.0 [8.0, 10.0]	8.0 [8.0, 8.0]	1.000
Extra‐PV targets	14.0 [12.0, 24.0]	15.0 [10.0, 26.0]	0.855
Total number of applications	34.0 [32.0, 40.0]	35.0 [32.0, 42.0]	0.129
Electrocardioversion during procedure	78 (33.8)	73 (37.1)	0.543
Vascular closure device (instead of figure of eight suture)	49 (21.2)	43 (21.8)	0.971
Protamine	79 (34.2)	45 (22.8)	0.013
Procedure time (min)	38.0 [32.0, 60.2]	55.5 [44.0, 107.8]	< 0.001
LA dwell time (min)	27.0 [23.0, 43.5]	45.5 [34.0, 88.2]	< 0.001
Fluoroscopy duration (min)	10.0 [8.0, 15.0]	12.0 [7.0, 17.0]	0.585

*Note:* Data is presented as counts (percentages) for categorical data, means ± standard deviations for normally distributed continuous data, and median [interquartile range] non‐normally distributed continuous data.

Abbreviations: ICE, intracardiac echocardiography; LA, left atrium; LCPV, left common pulmonary vein; LIPV, left inferior pulmonary vein; LSPV, left superior pulmonary vein; PVs, pulmonary veins; RMPV, right middle pulmonary vein; RIPV, right inferior pulmonary vein; RSPV, right superior pulmonary vein; TEE, transesophageal echocardiogram.

### 3.3 12‐Month Efficacy Outcomes

Thirteen patients (pentaspline catheter group: *n* = 8, circular catheter group: *n* = 5) were lost to follow‐up during the blanking period and were excluded from the 12‐month efficacy analyses. The median follow‐up duration was 364 days in both treatment groups, and follow‐up was performed with the use of a standardized home monitoring program in 50.8% of patients, interrogation of existing CIEDs in 8.0%, repeated Holter monitoring recordings in 8.2%, ECGs during outpatient clinic visits in 32.8%, and smartwatch measurements in 0.2%.

**Figure 1:**
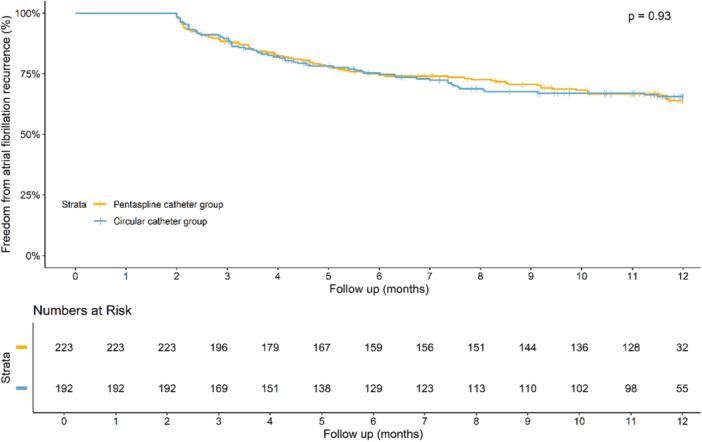
Kaplan−Meier curve: Recurrence of atrial fibrillation by ablation method. Kaplan−Meier curve illustrating recurrence of atrial fibrillation by ablation method.

**Figure 2:**
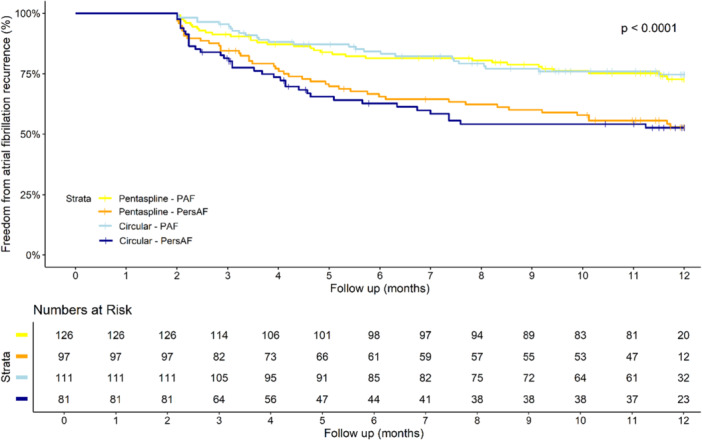
Kaplan−Meier curve: Recurrence of atrial fibrillation by ablation method and type of AF. Kaplan–Meier curves showing the recurrence of atrial fibrillation according to the ablation method and type of atrial fibrillation (AF). AF, atrial fibrillation; PAF, paroxysmal AF; PersAF, persistent AF.

Recurrence of AF after PVI beyond the blanking period was observed in 33.5% of patients at 12‐months, and recurrence of any atrial arrhythmia (including AF and atrial tachycardia) in 37.8% (Figure 1). Patients with paroxysmal AF showed lower AF recurrence rates after PVI compared to patients with persistent AF in both treatment groups; Pentaspline catheter group: 31.0% versus 48.5% (*p *= 0.012) and circular catheter group: 27.0% versus 50.6% (*p *= 0.001) (Figure 2). Rates of atrial arrhythmia recurrences were similar for both treatment groups: Pentaspline catheter group 38.6% versus circular catheter group: 37.0%, log‐rank *p* = 0.83. Univariate Cox regression showed that AF recurrence rates were higher in patients with an older age (HR 1.03, 95% CI 1.01−1.05, *p* = 0.003), persistent AF (HR 2.16, 95% CI 1.54−3.03, *p* < 0.001), longer time since diagnosis of AF (HR 1.04, 95% CI 1.00−1.07, *p* = 0.034), if AF(L) was present at start of the procedure (HR 2.02, 95% CI 1.44−2.83, *p* < 0.001), higher CHA₂DS₂‐VA‐score (HR 1.14, 95% CI 1.02−1.27, *p* = 0.017) and larger atrial volumes (LAVI: HR 1.03, 95% CI 1.01−1.05, *p*= 0.004). Multivariable Cox‐regression analysis showed that a longer time since AF diagnosis was the only variable associated with AF recurrence (HR 1.06, 95% CI 1.02−1.110) in patients with complete cases (*n* = 216). Multivariable analysis without LAVI (missing in > 25% of the cohort) was performed, showed that that persistent AF (HR 1.59, 95% CI 1.08−2.37, *p* = 0.02) was associated with AF recurrence. Both univariate and multivariable analyses showed no association between the ablation system and recurrence of AF. The results of the Cox regression analysis are presented in Table 3, whereas a more comprehensive comparison of baseline and procedural characteristics between patients with and without AF recurrence is provided in Supporting Information S1: Table 2.

**Table 3:** Univariate and multivariable Cox regression analyses—AF recurrence.

Variable	Univariate analysis	*p* value	Model 1: Complete case regression (*n* = 216)	*p* value	Model 2: Analysis without LAVI (*n* = 405)	*p* value
Age, years	HR 1.03 (95% CI: 1.01−1.05)	0.003	HR 1.02 (95% CI: 0.997−1.06)	0.080	HR 1.02 (95% CI: 0.99−1.05)	0.099
Female sex	HR 1.16 (95% CI: 0.82−1.64)	0.397				
BMI, kg/m^2^	HR 1.01 (95% CI: 0.98−1.04)	0.614				
Time since AF diagnosis, years	HR 1.04 (95% CI: 1.00−1.07)	0.034	HR 1.06 (95% CI: 1.02−1.11)	0.009	HR 1.03 (95% CI: 1.00−1.06)	0.086
Type of AF—Persistent	HR 2.16 (95% CI: 1.54−3.03)	< 0.001	HR 1.23 (95% CI: 0.81−2.01)	0.404	HR 1.60 (95% CI: 1.08−2.37)	0.020
CHA₂DS₂‐VA‐score	HR 1.14 (95% CI: 1.02−1.27)	0.017	HR 0.89 (95% CI: 0.73−1.10)	0.287	HR 1.03 (95% CI: 0.89−1.20)	0.685
LAVI, mL/m^2^	HR 1.03 (95% CI: 1.01−1.05)	0.004	HR 1.02 (95% CI: 1.00−1.04)	0.067		
Electroanatomic mapping system	HR 0.80 (95% CI: 0.57−1.12)	0.200				
Use of ICE periprocedural	HR 1.16 (95% CI: 0.82−1.65)	0.392				
Anesthesia—General anesthesia	HR 1.38 (95% CI: 0.90−2.13)	0.142				
PVI‐only procedure	HR 0.83 (95% CI: 0.53−1.29)	0.403				
Total number of applications	HR 1.00 (95% CI: 0.98−1.29)	0.657				
Rhythm at start of procedure—AF/AFL	HR 2.02 (95% CI: 1.44−2.83)	< 0.001	HR 1.37 (95% CI: 0.83−2.25)	0.216	HR 1.45 (95% CI: 0.98−2.16)	0.063
Ablation system PulseSelect	HR 0.99 (95% CI: 0.70−1.38)	0.932	HR 1.42 (95% CI: 0.91−2.21)	0.124	HR 0.95 (95% CI: 0.67−1.34)	0.757

Abbreviations: AF, atrial fibrillation; AFL, atrial flutter; BMI, body mass index; CI, confidence interval; HR, hazard ratio; ICE, intracardiac echocardiography; LAVI, left atrial volume index; PVI, pulmonary vein isolation.

### 3.4 Medication During Follow‐Up

Information regarding antiarrhythmic drug use at 12 months was available for a total of 326 patients (pentaspline catheter group: *n* = 175/223 [78.5%]; circular catheter group: *n* = 140/192 [72.9%]). The majority of patients who were treated with either class I or III anti‐arrhythmic drugs before the procedure were able to discontinue therapy within 1‐year post‐procedure (72.9%). β‐Blockers were often continued during follow‐up (61.9%). At 12 months of follow‐up, 19.0% of patients received treatment with class I or III anti‐arrhythmic drugs, compared to 52.7% pre‐procedural, with no significant differences between both groups (pentaspline catheter group: 12 months 18.9% vs. pre‐PVI 63.4%; circular catheter group: 12 months 19.3% vs. pre‐PVI 39.3%).

### 3.5 Repeat Ablation

**Figure 3:**
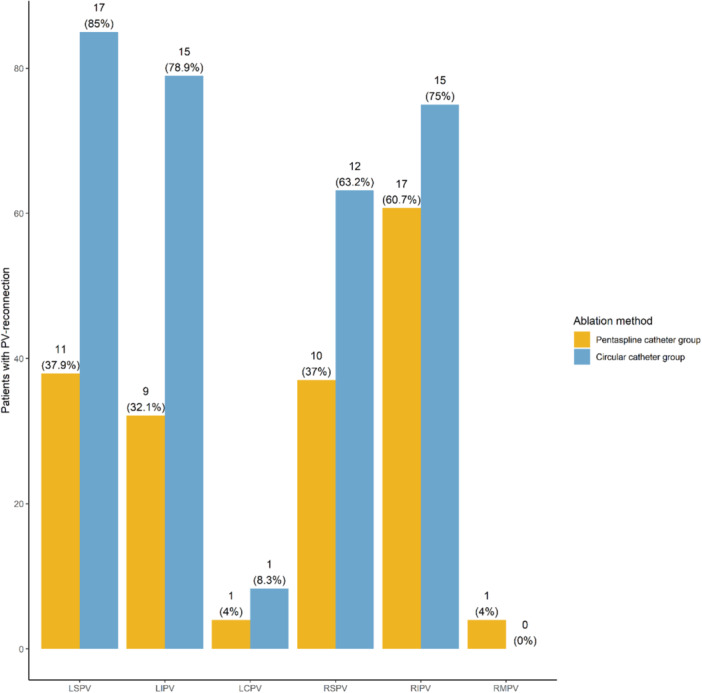
Reconnection of pulmonary veins during repeat ablation. Frequency of pulmonary vein reconnection by ablation method (pentaspline catheter‐group = yellow; circular catheter group = blue). Bars represent the proportion of patients with reconnection of each pulmonary vein displayed on the *X*‐axis, shown as the number of patients (*n*) and percentage (%). LCPV, left common pulmonary vein; LIPV, left inferior pulmonary vein; LSPV, left superior pulmonary vein; PV, pulmonary vein; RIPV, right inferior pulmonary vein; RMPV, right middle pulmonary vein; RSPV, right superior pulmonary vein.

During follow‐up, 32 patients (14.3%) of the pentaspline catheter group and 30 patients (15.6%) of the circular catheter group underwent a repeat ablation procedure (*p *= 0.762). Median time to repeat procedure was 222 days [166; 290] with no differences between treatment‐groups, *p* = 0.778. Patients previously treated with the pentaspline catheter had reconnection of a median of 2 [2; 3] veins during repeat procedure, with five patients (15.6%) showing durable isolation of all treated PVs. The RIPV was most often reconnected in these patients: *n* = 17 (60.7%). In the circular catheter group, the median number of veins showing reconnection was 3 [2.25; 4], which was comparable to the pentaspline catheter group (*p* = 0.069). Four patients (13.3%) had durable isolation of all PVs during the repeat ablation. At the PV level, reconnection of the LSPV and LIPV occurred significantly more frequently in the circular catheter group than in the pentaspline catheter group (LSPV: 85.0% vs. 37.9%, *p* = 0.003; LIPV: 78.9% vs. 32.1%, *p* = 0.004). The distribution of reconnection sites is presented in Figure 3.

### 3.6 Adverse Events

Major adverse events within 30 days post‐procedure included one patient with permanent phrenic nerve paralysis in the circular catheter group. In the pentaspline catheter group, one patient had a transient ischemic attack (TIA) post‐procedure, and one patient had a major access site bleeding complication in need of surgical intervention. A detailed description of all major adverse events is presented in Supporting Information S1: Table 3. Minor bleeding complications occurred more often in the pentaspline catheter group (sheath outer diameter 16.8 Fr) compared to the circular catheter group (sheath outer diameter 14.0 Fr): *n* = 28 (12.1%) versus *n* = 4 (2.0%), *p* < 0.001. During the 1‐year follow‐up, two patients in the circular catheter group died due to non‐cardiovascular causes.

## 4 Discussion

In our study, we compare long‐term efficacy outcomes of two commercially available PFA systems in performing PVI for AF. Both PFA systems were used side‐by‐side in all centers, and there was no specific patient selection. While there was no randomized selection process, the baseline patient characteristics of the two cohorts were similar. This study is one of the first to directly compare the long‐term efficacy of two widely used ablation systems, showing no difference in freedom from arrhythmias, both for paroxysmal as well as persistent AF patients.

PFA has rapidly become a widely adopted ablation technique for AF in routine clinical practice worldwide, and several PFA systems have been developed in recent years. While the underlying mechanism of all PFA systems is based on electroporation, variations in electric pulse parameters can influence the cell death mechanism and the subsequent formation of fibrosis [9]. Furthermore, differences in catheter design and the recommended number of applications might influence scar formation and consequently long‐term efficacy outcomes including arrhythmia‐free survival and the need for repeat ablation procedures [11].

Single‐arm studies regarding short‐term efficacy outcomes of the different PFA systems have shown promising results, with short procedure times, steep learning curves, high acute procedural success rates, and the ability to prevent common complications associated with thermal ablation systems [8, 12, 13]. Our observations further underline these promising short‐term efficacy outcomes, demonstrating acute procedural success in all patients, a low rate of major adverse events, and short procedure and LA‐dwell times, particularly in patients treated with the pentaspline catheter. Short procedure and LA‐dwell times may offer clinically relevant advantages through reduced anesthesia exposure and a potential reduction in thrombotic risks. However, data on long‐term efficacy outcomes remain limited. While research on the pentaspline‐catheter PFA system has shown similar to possibly even better long‐term outcomes for freedom of arrhythmias compared to thermal‐based PVI, randomized trials comparing different PFA systems are lacking [14, 15]. Recurrent atrial arrhythmias occurred in 37.3% of our study population during 1‐year follow‐up, following a 2‐month blanking period. Previous—mostly industry‐sponsored—(pre‐)clinical studies evaluating long‐term efficacy of various PFA‐systems have reported 12‐month atrial arrhythmia free survival rates between 15.5% and 32.3% after a 3‐month blanking period, with better long‐term efficacy in patients with paroxysmal AF compared to persistent AF [16, 17, 18]. In our cohort, only a minority of patients (15.4%) underwent a PVI‐plus procedure, with the posterior wall being the most frequently targeted area outside the pulmonary veins. Treatment of these regions did not appear to be associated with lower AF recurrence rates for both the pentaspline and circular catheter group in multivariable Cox regression analysis, consistent with previously published data [19].

To date, only one publication has compared 1‐year efficacy outcomes across different commercially available PFA systems [20]. Messori et al. applied an artificial intelligence algorithm to reconstruct patient‐level data from Kaplan−Meier curves of previously published studies evaluating three PFA systems: the FARAWAVE pentaspline catheter, the PulseSelect circular catheter, and the VARIPULSE circular catheter. This analysis was, however, limited by the predominantly single‐arm design of the included studies, the exclusion of patients with persistent AF, and the fact that only outcomes from the pentaspline catheter were presented in more than one included study. Nonetheless, they found no statistically significant differences between freedom from atrial arrhythmia recurrence rates between the evaluated systems. This finding is comparable to our results, that showed no difference in arrhythmia recurrence rates between treatment groups in a direct comparison of the two PFA systems used side‐by‐side in the participating centers.

Due to the retrospective design of our study—without formal randomization—and different institutional standards for the including centers, procedural characteristics showed marked variations between treatment groups, for example, the use of intra‐procedural echocardiography and 3D‐mapping systems. This heterogeneity might have influenced our outcomes and prevented a true head‐to‐head comparison. However, after correcting for these variables, multivariable Cox regression revealed no association between the PFA system and AF recurrences. Additionally, the regression model showed that time since the diagnosis of AF and type of AF were independently associated with AF recurrence in our cohort, which is in line with findings from previous publications [1, 21]. These findings support existing evidence for catheter ablation as a first‐line rhythm‐control strategy, suggesting that earlier intervention may improve long‐term outcomes [22, 23].

Arrhythmia recurrence rates are often reported as primary outcome parameter after PVI, but it is good to acknowledge the fact that not all documented arrhythmia recurrences are symptomatic. While the recurrence rate and time to first recurrence offer important information, these parameters do not account for the burden of symptoms, which is an important predictor for quality of life and repeat ablation procedures [24]. In this study, 14.8% of patients underwent a repeat ablation procedure due to recurrent atrial arrhythmias within 1 year after the index PVI, which is comparable to rates found in previous published studies [25, 26, 27, 28]. Of this cohort with arrhythmia recurrence, only a minority of patients (13.6%) had durable isolation of all PVs at the redo procedure, which is lower than the wide range of previously reported percentages of durable isolation after PFA from clinical studies, with percentages between 18.2% and 76.0% [25, 26, 27, 29, 30]. When comparing repeat ablation outcomes for the different PFA‐systems, patients treated with the circular catheter had a (nonsignificant) higher number of non‐isolated PV's compared to patient treated with the pentaspline catheter, especially apparent for the left PV's. We hypothesize that these differences might be due to the learning curve of operators, catheter positioning to cover the complete circumference of the antrum, tissue‐contact stability, and variations in catheter design, with a possible higher risk of non‐overlapping applications with the smaller circular catheter (diameter 25 vs. 31 mm for the pentaspline catheter) and a consequent higher rate of gaps in isolation. Although imaging was more frequently used with the circular catheter, its impact on gaps could not be reliably assessed due to the relatively low number of repeat ablations. Furthermore, the clinical relevance of the number of reconnected veins remains unclear, as not all pulmonary veins are inherently arrhythmogenic, and reconnections may not necessarily result in recurrent AF [31].

## 5 Limitations

This study has several limitations that should be considered with the interpretation of the results. First, the study is limited by the retrospective, observational design leading to missing data and the possibility to induce bias. Secondly, the design precludes formal randomization of patients between treatment groups. While the selection of the PFA system for each patient was primarily based on system availability with no formal selection criteria, some selection bias cannot be completely excluded, which may have influenced outcomes. Nevertheless, given the current challenges of conducting adequately powered randomized trials on this topic, this multicenter registry provides the most feasible and informative real‐world comparison currently available. Lastly, due to the multicenter design of this study, marked differences in peri‐ and post‐procedural management exist between centers. For example, the periprocedural use of ICE guidance, ablation of extra‐PV targets, and electroanatomic mapping are greatly institutionally dependent. However, when performing multivariable regression analyses, these variables did not seem to influence long‐term efficacy outcomes. Although differences in follow‑up strategies between centers could affect the detection of recurrent atrial arrhythmias, these strategies were center‑based rather than system‑based and therefore unlikely to systematically favor one ablation system over the other. We believe that the multicenter design offers additional value to a single‐center design in terms of a larger sample size, higher generalizability, and providing a real‐world overview of outcomes with centers in Europe, the USA, and Australia. Nonetheless, additional studies with formal randomization and strict protocols are needed to support our findings.

## 6 Conclusion

This multicenter study is one of the first to report a comparison of 12‐month efficacy outcomes of two different PFA systems in a large, real‐world, all‐comers patient population. The results showed no differences in atrial arrhythmia recurrence rates, repeat ablation rates at 12 months, or major adverse event rates.

## Funding

The authors have nothing to report.

## Conflicts of Interest

J.C.B. reports being a consultant/proctor for Abbott. S.M. has served as a consultant for and participated in PFA clinical trials sponsored by Boston Scientific and Medtronic. B.P.K. has served as a consultant for and participated in PFA clinical trials sponsored by Abbott, Boston Scientific, and Medtronic. M.v.d.G. reports receiving speaker's honoraria, travel grants, and consulting honoraria from Abbott, Biosense Webster, Boston Scientific/Farapulse Inc, Medtronic, Lumavision, Biotronik, Bristol‐Myers Squibb, and Zoll. B.W. reports being a speaker/advisor for Medtronic and Boston Scientific. T.H. reports receiving speakers' honoraria for Abbott and an educational grant from Boston Scientific. M.L. reports being a consultant/proctor for Medtronic and Abbott. D.L.M. reports being a consultant for AltaThera Pharmaceuticals and SentiAR, and participated in trials sponsored by Boston Scientific and Medtronic. M.C.E.F.W. reports being a speaker for Abbott. V.F.v.D. reports being a consultant for Medtronic and Boston Scientific. L.V.A.B. reports being a consultant/speaker/proctor for Boston Scientific and Medtronic and a speaker for ZOLL, Biosense Webster, and Abbott. All fees go to the cardiology department. The other authors declare no conflicts of interest.

## Supporting information


**Table S1:** Multivariable linear analysis for total procedure time and LA‐dwell time. **Table S2:** Differences in baseline, procedure and follow‐up variables for patients with and without AF recurrences at 1 year. **Table S3:** Description of major adverse events during follow‐up.

## Data Availability

The data that support the findings of this study are available from the corresponding author upon reasonable request.
