# Supplementary material for: Comparison of Two Pulsed Field Ablation Systems for Atrial Fibrillation: One‐Year Outcomes From a Multicenter Registry
Source: J Cardiovasc Electrophysiol. 2026 May 26;37(7):1522–32. doi: 10.1111/jce.70375 (PMC13372399; doi:10.1111/jce.70375)
Supplement: Supplementary file 1 — Table S1: Multivariable linear analysis for total procedure time and LA‐dwell time. Table S2: Differences in baseline, procedure and follow‐up variables for patients with and without AF recurrences at 1 year. Table S3: Description of major adverse events during follow‐up. [file JCE-37-1522-s001.pdf]

SUPPLEMENTAL MATERIAL

Supplementary Table 1: Multivariable linear analysis for total procedure time and LA-dwell time

	**Total procedure time**<br>*Intercept = 31.5 min*		**LA-dwell time**<br>*Intercept = 21.0 min*	
**Variable**	**Procedure time Δ %**	**p-value**	**LA-dwell time Δ %**	**p-value**
BMI	+0.2%<br>(95% CI: -0.5% – 0.8%)	0.630	+0.3%<br>(95% CI: -0.4% – 0.9%	0.454
Anesthesia –<br>General anesthesia	+25.1%<br>(95% CI: 15.5 – 35.5%)	<0.001	+28.8%<br>(95% CI: 17.9% – 40.8%)	<0.001
PFA-method –<br>Circular catheter	+17.5%<br>(95% CI: 10.6% – 25.0%)	<0.001	+26.7%<br>(95% CI: 18.3% – 35.7%)	<0.001
PVI-only procedure	-12.2%<br>(95% CI: -20.2% – -3.3%)	0.008	-13.1%<br>(95% CI: -21.8% – -3.5%)	0.009
Use 3D mapping system	+58.0%<br>(95% CI: 41.1 – 77.0%)	<0.001	+77.3%<br>(95% CI: 56.0% – 102%)	<0.001
Use of echocardiography – TEE	-7.1%<br>(95% CI: -19.2% – 6.9%)	0.302	-3.0%<br>(95% CI: -16.8% – 13.2)	0.703
Use of echocardiography – ICE	+76.5%<br>(95% CI: 56.8% – 98.6%)	<0.001	+67.2%<br>(95% CI: 46.7% – 90.7%)	<0.001
Electrocardioversion during procedure	+1.4%<br>(95% CI: -4.5% – 7.5%)	0.653	+3.1%<br>(95% CI: -3.5% – 10.1%)	0.365

Abbreviations: BMI: Body Mass Index; PFA: Pulsed field ablation; ICE: Intracardiac Echocardiography; PVI: Pulmonary Vein Isolation; TEE: Transesophageal Echocardiogram.

Supplementary Table 2: Differences in baseline, procedure and follow-up variables for patients with and without AF recurrences at 1 year

	No AF recurrence (n=276)	AF recurrence (n=139)	p-value
Female sex	88 (31.9)	51 (36.7)	0.385
Age, years	62.0 ± 10.7	65.3 ± 9.2	0.001
BMI, kg/m2	27.9 ± 5.0	28.3 ± 4.8	0.486
Hypertension	105 (38.0)	61 (43.9)	0.298
Diabetes mellitus	28 (10.1)	17 (12.2)	0.633
Heart failure	34 (12.3)	17 (12.2)	1.000
OSA	41 (14.9)	21 (15.1)	1.000
CHA_2DS_2-VA-score	1.4 ± 1.4	1.7 ± 1.5	0.027
LAVI, mL/m^2 (n=220)	31.2 ± 9.6	36.1 ±11.0	<0.001
LVEF <40% (n=309)	7 (3.5)	4 (3.7)	1.000
Type of AF			<0.001
Paroxysmal AF	178 (64.5)	59 (42.4)	
Persistent AF	95 (34.4)	79 (56.8)	
Longstanding persistent	3 (1.1)	1 (0.7)	
Time since AF diagnosis, years	1.5 [0.5, 4.2]	2.2 [0.8, 5.2]	0.017
Use of beta blockers pre-procedural	123 (44.6)	75 (53.5)	0.153
Use of class I or III antiarrhythmic drugs pre-procedural	143 (51.8)	75 (54.0)	0.757
Treatment group			0.866
Pentaspline catheter group	147 (53.3)	76 (54.7)	
Circular catheter group	129 (46.7)	63 (45.3)	
General anesthesia (instead of conscious sedation)	210 (76.1)	113 (81.3)	0.280
Use of echocardiography – TEE	21 (7.6)	6 (4.3)	0.283
Use of echocardiography – ICE	88 (31.9)	49 (35.3)	0.563
Electroanatomic mapping system	128 (46.4)	57 (41.0)	0.350
PVI-only procedure	239 (86.6)	116 (83.5)	0.477
Total number of applications	34.0 [32.0, 40.0]	35.0 [32.0, 40.0]	0.762
Electrocardioversion during procedure	74 (26.8)	70 (50.4)	<0.001
Procedure time (min)	46.0 [36.0, 90.0]	47.0 [34.8, 86.5]	0.959

Use of beta-blockers at 1 year post-procedure	57 (27.7)	46 (42.2)	0.013
Use of class I or III antiarrhythmic drugs at 1 year post-procedure	20 (9.7)	40 (36.7)	<0.001
Follow-up method			0.016
Homemonitoring program	134 (48.6)	77 (55.4)	
ECGs during visits	100 (36.2)	36 (25.9)	
Interrogation of CIED	16 (5.8)	17 (12.2)	
Holter monitoring	26 (9.4)	8 (5.8)	
Smartwatch	0 (0.0)	1 (0.7)	

Data is presented as counts (percentages) for categorical data, means ± standard deviations for normally distributed continuous data and median [interquartile range] non-normally distributed continuous data.

Abbreviations: AF: Atrial Fibrillation; BMI: Body Mass Index; CIED: Cardiac Implantable Electronic Device; ICE: Intracardiac Echocardiography; LAVI: Left Atrial Volume Index; LVEF: Left Ventricular Ejection Fraction; OSA: Obstructive Sleep Apnea; PVI: Pulmonary Vein Isolation; TEE: Transesophageal Echocardiogram.

Supplementary Table 3: Description of major adverse events during follow-up

Treatment group	Patient	Complication	Event description
Circular catheter group	Female 78y<br><br>Hypertension, COPD, OSA, paroxysmal AF, BMI 28.6	Phrenic nerve paralysis	The procedure included a PVI-only procedure with the use of a 3D-mapping system and ICE. A total of 35 applications were given. Date: May 6, 2024<br><br>During the procedure, no phrenic nerve paralysis was observed. However, at a follow-up visit on June 6, 2024, the patient reported shortness of breath. Chest radiography performed the same day revealed an elevated right hemidiaphragm, which remained persistent as of May 2025. No imaging was performed prior to the procedure.
Pentaspline catheter group	Female 59y<br><br>Paroxysmal AF, BMI 29.1<br><br>Med: Uninterrupted Edoxaban	TIA	The procedure included a PVI-only procedure with a total of 34 applications. Date: March 21, 2024<br><br>The patient experienced homonymous hemianopsia for a total of 2 hours on day 3 post-procedure. A CT- scan revealed no ischemic lesions or other anomalies. No MRI was performed. Given the timing of the event, this event may be attributable to a late embolism following ablation.
Pentaspline catheter group	Female 60y<br><br>Hypertension, hyperlipidemia, paroxysmal AF, BMI 30.5<br><br>Med: Uninterrupted Edoxaban	Major access site bleeding	The procedure was a PVI-only procedure, without the use of a 3D-mapping system of echocardiographic imaging. Femoral closure with manual pressure and figure-of-8 suture, protamine was given. Date: January 29, 2024<br><br>After the procedure, bleeding occurred from the right superficial femoral artery. Hemostasis was achieved with thrombin injection. However, ultrasound on January 31 revealed a persistent pseudoaneurysm and arteriovenous fistula, requiring percutaneous transluminal angioplasty of the superficial femoral artery with placement of a covered stent.

Abbreviations: AF: Atrial Fibrillation; BMI: Body Mass Index; COPD: Chronic Obstructive Pulmonary Disease; CT: Computed Tomography; ICE: Intracardiac Echocardiography; MRI: Magnetic Resonance Imaging; OSA: Obstructive Sleep Apnea; PVI: Pulmonary Vein Isolation; Med: Medication.
